# Enhanced Performance of Artificial-Neural-Network-Based Equalization for Short-Haul Fiber-Optic Communications

**DOI:** 10.3390/s23135952

**Published:** 2023-06-27

**Authors:** Mahmoud M. T. Maghrabi, Hariharan Swaminathan, Shiva Kumar, Mohamed H. Bakr, Shirook M. Ali

**Affiliations:** 1 Department of Electrical and Computer Engineering, McMaster University, Hamilton, ON L8S 4K1, Canada; swaminah@mcmaster.ca (H.S.); skumar@mcmaster.ca (S.K.); mbakr@mcmaster.ca (M.H.B.); 2 Department of Engineering Mathematics and Physics, Cairo University, Giza 12613, Egypt; 3 School of Mechanical and Electrical Engineering Technology, Sheridan College, Brampton, ON L6Y 5H9, Canada; shirook.ali@ieee.org

**Keywords:** artificial neural network (ANN), digital signal processing (DSP), fiber-optic communications, intensity modulation and direct detection (IMDD), short reach

## Abstract

This work proposes an efficient and easy-to-implement single-layer artificial neural network (ANN)-based equalizer with improved compensation performance. The proposed equalizer is used for effectively mitigating the distortions induced in the short-haul fiber-optic communication systems based on intensity modulation and direct detection (IMDD). The compensation performance of the ANN equalizer is significantly improved, exploiting an introduced advanced training scheme. The efficiency and robustness of the proposed ANN equalizer are illustrated through 10- and 28-Gbaud short-reach optical-fiber communication systems. Compared to the efficient but computationally expensive maximum likelihood sequence estimator (MLSE), the proposed ANN equalizer not only significantly reduces its computational equalization cost and storage memory requirements, but it also outperforms its bit error rate performance.

## 1. Introduction

Optical fibers are widely deployed in modern telecommunication systems and networks [1,2]. They have been used in several short-reach applications [3]. Metro and media access networks, data center networks (DCNs), cloud radio access networks (C-RANs), passive optical networks (PONs), and mobile front-haul networks (MFHNs) are a few examples among many other potential applications of short-haul fiber-optic communications [4,5,6].

In short-reach applications, intensity modulation and direct detection (IMDD) is typically used because of its low cost, simple implementation, and robustness [7]. The nonlinear Kerr effect is negligible in the short-haul fiber-optic transmission systems due to small launch power and short transmission range. However, linear optical distortions caused by the unavoidable chromatic dispersion phenomenon of the fiber turn into nonlinear impairments in the electrical domain due to the square-law detection of the IMDD system. Therefore, linear electrical equalizers, e.g., feed-forward equalizer (FFE) [8] and decision feedback equalizer (DFE) [9], fail to mitigate such nonlinear signal distortions at the receiver. Rather, a nonlinear equalizer has to be employed at the receiver side to perfectly compensate for the nonlinear distortions.

Various nonlinear electronic equalization techniques have been proposed in the literature for mitigating the fiber distortions of the IMDD fiber communication systems [10]. For instance, maximum likelihood sequence estimator (MLSE) is the classical technique that provides the traditional benchmark compensation performance [11,12]. However, it suffers from its high computational cost that prohibits its practical implementation. The MLSE computational cost per symbol is exponentially proportional to the length of the intersymbol interference (ISI) span, i.e., the required channel memory size. The high MLSE computational cost can though be reduced at the expense of lower equalization performance using a Volterra-series-based equalizer (VSE) [13,14]. However, the computational cost of VSEs is still unreasonably high because of the enormous amount of calculations required, especially when a high transmission bit rate is needed.

A powerful nonlinear equalization technique that can potentially reduce the computational complexity required by the VSE is a neural-network-based equalizer (NNE) [15]. While the computational cost of the NNEs increases in proportion to the number of neurons in each layer, the VSE cost increases exponentially as the number of tapped delays and the order of the Volterra series increase [16,17,18]. Particularly, in order to compensate for the nonlinear impairments of the short-reach fiber transmission system, a computational cost per symbol of as low as $O\left(N^{2}\right)$ is required by the NNE, where N is the ISI span length [16,17,18]. Contrarily, the minimum computational complexity of the VSE is of the order $O\left(N^{3}\right)$ per symbol [16,17,18].

Several types of NNEs have therefore been exploited for the compensation of the electrical nonlinear impairments in the IMDD short-haul fiber-optic communication systems. These include equalizers that are based on single-layer artificial neural networks (ANNs) [19,20,21], two-layer ANNs [22,23,24], radial basis function neural networks [25], convolutional neural networks [23,26,27], recurrent neural networks [28,29], and multi-layer deep neural networks [6,30]. Although the single-layer-ANN-based equalizers are the most basic option among all other types of NNEs, they are more popular to be practicably utilized in the short-reach fiber-optic applications because of their superiority from the computational cost point of view. In [21], we proposed an extensive training scheme that enhances the compensation performance of the single-layer-ANN-based equalizer, achieving a slightly lower compensation performance compared to that of the MLSE but with much lower computational cost. Instead of utilizing typical random training data, the extensive training scheme uses a set of data that is properly chosen according to the physical nature of the fiber-induced signal distortions.

In this paper, we propose a more efficient ANN equalizer for mitigating the nonlinear electrical impairments induced in the IMDD short-reach optical-fiber communication system. The proposed equalizer consists of a basic single-hidden-layer ANN that minimizes the overhead computational complexity required for the signal recovery at the receiver end. An advanced training method is also introduced to significantly improve the bit error rate (BER) performance of the equalizer, compared to the ANN equalizer introduced in [21], with the same computational cost per symbol. The proposed training scheme considers multiple noisy sets of all possible data combinations that could be transmitted through the optical channel. This is contrasted with the extensive training scheme introduced in [21], which utilizes only noise-free data during the training process. The robustness, performance, and efficiency of the proposed ANN equalizer are demonstrated through applying it to mitigate the distortions of 10- and 28-Gbaud short-reach optical-fiber communication scenarios with the IMDD system. Thanks to the advanced training scheme, the compensation performance of the proposed ANN equalizer outperforms the BER performance obtained using the robust but computationally expensive MLSE equalizer.

The remainder of the paper is organized as follows. Section 2 introduces the operation principles of the proposed ANN-based equalization technique. A full description of the introduced training scheme is also provided. In Section 3, we present system setups, results, and discussions on the evaluation of system BER performance, computational complexity, and efficiency. Finally, conclusions of the work are drawn in Section 4.

## 2. Principles of the Proposed ANN Equalizer

Linear optical distortions induced in the short-haul fiber-optic link are converted into nonlinear impairments in the electrical domain. This is because of losing the phase information due to the square-law detection of the direct detector (DD). A nonlinear electrical equalizer is therefore needed at the digital signal processing (DSP) unit to compensate for the nonlinear distortions, recovering the transmitted data. Neural networks have shown promising modeling performance in various biomedical and chemical engineering, biological, and medical fields and applications [31,32]. Due to the superior linear and nonlinear modeling ability of artificial neural networks (ANNs), they can be utilized, after a proper training scheme, to compensate for these nonlinear system distortions, inverting the response of both the fiber channel and direct photo-detection system.

**Figure 1:**
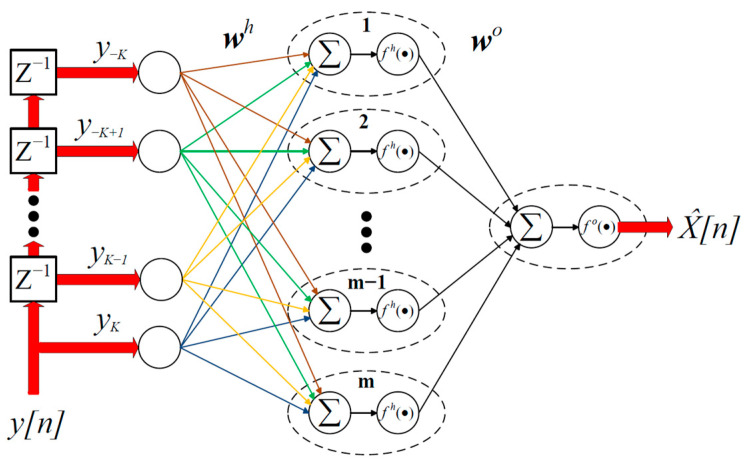
Structure of the proposed artificial neural network (ANN)-based equalizer.

The schematic of the proposed ANN nonlinear equalizer is shown in Figure 1. Since our objective is to implement an effective but computationally inexpensive nonlinear equalizer, we select the basic ANN structure consisting of an input layer, a single hidden layer, and an output layer. The input layer comprises N nodes, each of which is triggered by a sample of the received (distorted) signal $y\left[n\right]$. In other words, the input vector at each time instant i represents the current input sample with its K/2 previous and K/2 subsequent samples, where N=2K+1 is the length of the intersymbol interference (ISI) span. Each line connecting two nodes represents a multiplier that multiplies the output of the previous layer by the corresponding weight. The hidden and output layers include m and 1 nodes, respectively. Each node in the hidden and the output layer is a computing unit that applies a nonlinear activation function to the summation of its weighted inputs. The relationship between the input vector $Y=\left[y_{-K}\;y_{-K+1}\;y_{-K+3}\cdots y_{K}\right]$ and the ANN output $\hat{X}$ is thus expressed as follows:

(1) $$\hat{X}=f_{o}\left(\sum_{j=1}^{m}w_{j}^{o}f_{h}\left(\sum_{l=1}^{N}w_{jl}^{h}\;y_{l}\right)\right),$$

where $w_{jl}^{h}$ is the weight assigned to the connection between the $l^{th}$ input $y_{l}$ and the $j^{th}$ node of the hidden layer. The weight $w_{j}^{o}$ is assigned to the connection between the $j^{th}$ hidden-layer node and the output-layer node. The scalar functions $f_{h}\left(\cdot \right)$ and $f_{o}\left(\cdot \right)$ are the nonlinear activation functions of the hidden and output layers, respectively. It should be clear that the ANN output $\hat{X}$ represents the equalized sample at time instant i. Using a proper training scheme, the neural network weights can be adjusted such that the output sequence $\hat{X}\left[n\right]$ represents an approximate estimate of the undistorted transmitted sequence $x\left[n\right]$.

The training of the ANN equalizer is performed as follows. A stream of known data is first transmitted through the fiber channel to the receiver, and a set of known input–output training data pairs is formulated. The weights of the ANN equalizers are then adjusted by recursively solving an optimization problem that minimizes the mean square error (MSE) between the equalizer outputs (representing the equalized signal samples) and the desired outputs (representing the transmitted signal samples). The training problem is thus given by

(2) $$\underset{W}{min}\;E\left(W\right)=\frac{1}{N_{tr}}\sum_{k=1}^{N_{tr}}\|{\hat{X}}_{k}\left(W,Y_{k}\right)-X_{k}\|{}_{2}^{2},$$

where $W={\left[W_{h}^{T}\;W_{o}^{T}\right]}^{T}$ is a vector that contains the set of weights connecting the input parameters to the hidden-layer nodes $W_{h}$ and the set of weights connecting the hidden-layer nodes to the output-layer node $W_{o}$. The operation ${\left\|\cdot \right\|}_{2}$ denotes Euclidean norm, $N_{tr}$ is the number of training symbols, and $Y_{k}$ is the input equalizer vector corresponding to the $k^{th}$ training symbol. The parameters ${\hat{X}}_{k}$ and $X_{k}$ are the equalizer output and the corresponding desired output for the $k^{th}$ training symbol, respectively.

To achieve the most efficient ANN equalization performance, converging to the global optimal weights of the equalizer, it is crucial to determine (i) the number of input-layer nodes N, i.e., the number of equalizer taps, (ii) the number of hidden-layer nodes m, and (iii) the number of training data $N_{tr}$ and the way to select them. The number of equalizer taps N is chosen according to the length of the ISI of the channel. In other words, depending on modulation format, optical-fiber length L, and dispersion parameter $\beta_{2}$ of the optical-fiber channel, the number of neighboring symbols K that may interfere with the current symbol (i.e., ISI span) is estimated. The number of taps required by the equalizer is then determined as N=2K+1. The number of required hidden nodes m is obtained through a convergence analysis. First, the hidden nodes number m is set to a certain small number, e.g., 3, and the training problem (2) is solved, and the terminated MSE is calculated. Then, we gradually increase the value of m and resolve problem (2) until the terminated MSE value saturates up to a certain accuracy.

**Figure 2:**
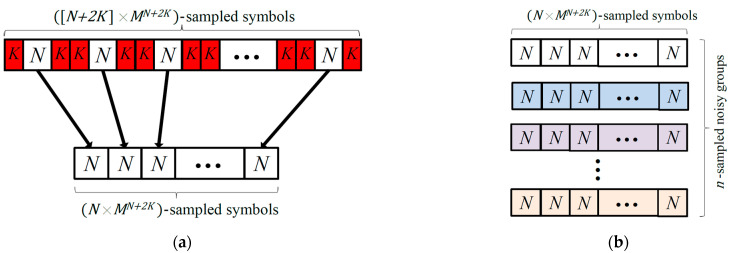
Schematic diagram of the training data preparation for an M-ary modulation format in the case of (**a**) extensive training scheme [21] and (**b**) advanced training scheme. K is the number of interfering symbols and N=2K+1 is the ISI span length, i.e., the required number of equalizer taps. The arrows refer to the original data used during the training of the ANN equalizer, after discarding the redundant guard data.

Typically, choosing the training data and their number $N_{tr}$ is done randomly. However, it has been shown in [21] that the equalization performance of neural networks-based equalizers could be significantly enhanced utilizing an extensive training scheme that considers all possible patterns to be received by the equalizer later on. Instead of transmitting a random dataset during the training phase, the extensive training scheme uses a complete set of training data formulated as in [21]. For an M-ary modulated data and ISI span length of N (i.e., K interfering symbols), there is a total combination of $M^{N}$ distinct patterns of the training data samples. In other words, we have $\left(N\times M^{N}\right)$ different input training vectors and $\left(1\times M^{N}\right)$ corresponding desired outputs. However, while propagating inside the fiber, the outer symbols in each pattern can be affected by the tail of its successive neighboring patterns. Hence, the equalizer may receive patterns that are not included in those basic $M^{N}$ patterns. To take into account all possible combination patterns that could be received, K redundant guard symbols are added around the sides of each basic pattern, as shown in Figure 2a. First, $M^{N+2K}$ different symbols are generated and transmitted through the fiber. At the DSP unit, the samples corresponding to those redundant guard symbols are discarded, as illustrated in Figure 2a, and only samples with regard to original data are used to train the equalizer. The number of training data used by the extensive training scheme is therefore $N_{tr}=M^{N+2K}$. In other words, a total of $\left(N\times M^{N+2K}\right)$ input training vectors and $\left(1\times M^{N+2K}\right)$ corresponding desired outputs are utilized. It should be clear that this extensive training scheme considers all possible received patterns but in a noise-free channel. In other words, it does not account for the noise induced in the system. Therefore, the equalization performance of an NN-based equalizer trained using the extensive training scheme is severely limited by the system noise. The equalization performance though may be potentially enhanced if noisy data are used to train the equalizer’s neural network.

To enhance the efficiency and robustness of the ANN equalizer, we propose an advanced training scheme that utilizes multiple noisy sets of the complete patterns used in the extensive training scheme. In particular, the training data of the advanced training scheme are obtained as follows. We first generate $\left(M^{N+2K}\right)$ different symbols, including $M^{2K}$ redundant guard symbols, similar to the case explained in the extensive training scheme. This symbol data stream is then transmitted through the optical-fiber system that adds noise, as well as distortions due to chromatic dispersion. The discretized received signal samples pass through the DSP unit. The samples corresponding to the redundant guard data are discarded (see Figure 2a) and the signal samples corresponding to the original training symbols and their transmitted peers are used to formulate the first group of input–output training pairs, as shown in Figure 2a. To take into account the channel noise, the same stream of data, consisting of $\left(M^{N+2K}\right)$ distinct symbol patterns, is resent through the fiber system n times. The same procedures are then repeated at the DSP unit to formulate a total of n different groups of input–output training pairs, each of which consists of $\left(N\times M^{N+2K}\right)$ input training vectors and $\left(1\times M^{N+2K}\right)$ corresponding desired outputs, as shown in Figure 2b. Note that the desired output vectors in all groups are identical; however, the input training vectors in each group have different values due to randomness of the system noise. The total number of training data needed by the advanced training scheme is $N_{tr}=n\times M^{N+2K}$. In the form of neural network training data, a total of $n\times \left(N\times M^{N+2K}\right)$ input training vectors and $n\times \left(1\times M^{N+2K}\right)$ corresponding desired output vectors are used.

Once the input–output training data pairs are determined, the optimization training problem (2) is solved using any well-defined optimization algorithm to obtain the optimal ANN weights $W^{*}$. In this work, we specifically use the adaptive moment estimation (Adam) algorithm [33,34]. The Adam algorithm is one of the most effective stochastic-gradient-based optimization algorithms that is widely used to solve training problems of ANNs and deep neural networks. It shows efficient ability to solve optimization problems with a large number of data and parameters, with little memory requirement. It also offers superior effectiveness to solve problems that include very noisy and sparse gradients, which is the common case in neural network training problems. The Adam algorithm is derived from the calculation of the evolutionary moment. It uses estimations of the first and second moments of the gradient to iteratively update weights of the neural network.

### Advanced Training Scheme Algorithm

**Figure 3:**
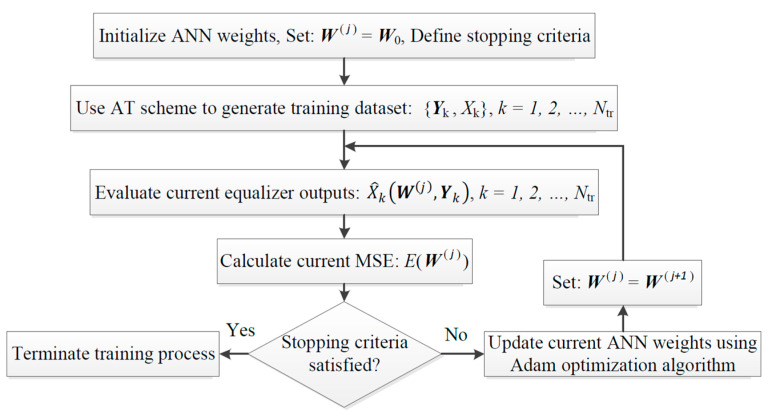
Flow diagram of the introduced advanced training scheme algorithm used to train the proposed ANN-based equalizer. ANN: artificial neural network; AT: advanced training; MSE: mean square error; Adam: adaptive moment estimation.

The flow diagram summarizing the training algorithm of the proposed ANN-based equalizer, utilizing the introduced advanced training scheme, is shown in Figure 3. First, the ANN weights are assigned to random initial values, and the stopping criteria of the training algorithm are defined (e.g., minimum required mean square error (MSE)). The maximum allowed number of training epochs and/or improvement of validation accuracy is determined. The advanced training scheme is then utilized to generate the ANN training input–output dataset, as illustrated in Figure 2b. Using the current weight values, the corresponding ANN equalizer outputs are evaluated, and the corresponding MSE between current equalizer outputs and desired outputs is calculated (see Equation (2)). The algorithm then checks whether the predefined stopping criteria are satisfied or not. In other words, it determines whether the current ANN model is well trained or not. If all stopping criteria are satisfied, the algorithm terminates, and the best-achieved ANN model is saved for testing. Otherwise, the Adam optimization algorithm is employed to update the current ANN weights such that the current MSE is reduced. The new ANN model (i.e., ANN with new weight parameters) is considered as the current model. The current equalizer outputs and MSE are re-calculated, and the cycle is repeated till the algorithm terminates.

Once the training of the ANN equalizer is completed, actual unknown data can then be transmitted through the channel, and the trained ANN equalizer is utilized at the DSP unit of the receiver to compensate for the system distortions, recovering the transmitted data. In this case, the input vector of the ANN equalizer at time instant i is given as

(3) $$\;Y_{i}={\left[y_{-K+i,\;\;\;}y_{-K+i+1}\;\ldots \;\;y_{i}\ldots \;y_{K+i-1},\;\;y_{K+i}\right]}^{T},\;\;\;i=K,\;K+1,\;K+2,\;\ldots ,$$

where $y_{i}$ is the received sampled symbol at time instant i, and K is the number of interfering symbols. For each input vector $Y_{i}$, the ANN equalizer produces an equalized signal sample ${\hat{X}}_{i}$, which is assumed to be a reliable estimate of the actual transmitted signal sample $X_{i}$.

Notice that during the transmission of actual data, the nominal parameters of the fiber channel (e.g., dispersion coefficient $\beta_{2}$) might be subjected to fluctuations due to variations in environmental conditions. Although these fluctuations occur at a much slower rate than the transmission data rate, it can slightly deteriorate the performance achieved by the trained ANN equalizer. However, the proposed ANN equalizer can be simply modified to adaptively re-adjust its weights and trace these channel fluctuations using a small variation in the weights’ values. In practice, only one optimization step is needed [2]. Since we have no information about the transmitted signal, in this case, the single optimization step is performed to minimize the MSE between the final decided symbol value ${\hat{a}}_{i}$ and the equalizer output ${\hat{X}}_{i}$, i.e., the objective function of Equation (2) becomes $E\left(W\right)={\left[{\hat{X}}_{i}\left(W,Y_{i}\right)-{\hat{a}}_{i}\right]}^{2}$.

## 3. Results and Discussion

**Figure 4:**
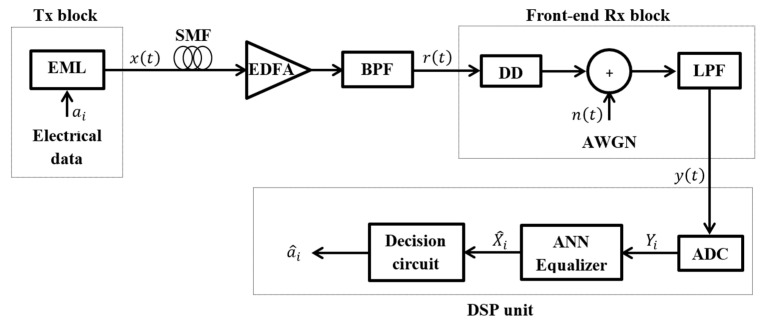
Block diagram of the intensity modulation and direct detection short-reach optical-fiber communication system setup considered in the simulation. Tx: transmitter; EML: electrically modulated laser; SMF: single-mode fiber; EDFA: erbium-doped fiber amplifier; BPF: band-pass filter; Rx: receiver; DD: direct detector; AWGN: additive white Gaussian noise; LPF: low-pass filter; ADC: analog-to-digital converter; ANN: artificial neural network.

Figure 4 shows the system setup used for evaluating the performance of the proposed ANN equalization method. At the transmitter side, independent and identically distributed input symbols $a_{i}$ are randomly generated. The data are mapped into an on–off keying (OOK) modulation format with a non-return-to-zero (NRZ)-raised cosine pulse shaping whose roll-off factor is 0.6. An electrically modulated laser (EML) is used to convert electrical signal into optical domain, obtaining the output transmitted optical signal $x\left(t\right)$. Two data rates are considered during the simulations, namely, a 10-Gbaud- and 28-Gbaud-rate system. The optical transmission channel is a standard linear single-mode fiber (SMF) whose nominal parameters are given as dispersion coefficient $\beta_{2}=-21\;{\mathrm{ps}}^{2}/\mathrm{km}$, loss coefficient α=0.2 dB/km, and length L in km. The SMF is followed by an inline erbium doped-fiber amplifier (EDFA) that fully compensates for the fiber loss. Unless otherwise stated, the noise of the EDFA is neglected. The output of the EDFA passes through an optical Gaussian band-pass filter (BPF) of bandwidth 50 GHz or 100 GHz at the 10-Gbaud- or 28-Gbaud-system, respectively.

At the receiver, a direct-detection (DD) receiver is first employed for optical-to-electrical conversion whose output is proportional to the square of the magnitude of the distorted optical received signal $r\left(t\right)$. The linear distortions of the chromatic dispersion in the optical domain are converted into nonlinear distortions in the electrical domain because of the square-law detection of the DD. In other words, the phase information of the received optical signal is lost in the electrical domain. A Gaussian low-pass filter is placed after the DD to reduce the noise. Its 3 dB bandwidth is 7 GHz or 21 GHz for the 10-Gbaud- or 28-Gbaud-system, respectively. Note that the additive white Gaussian noise $n\left(t\right)$ added by the DD consists of shot and thermal noise [1,2]. At the DSP unit, an analog-to-digital converter (ADC) is used to reduce the sampling rate from 16 samples per symbol to 2 samples per symbol, which is sufficient for accurate reconstruction of time-continuous signals according to the Nyquist condition. The proposed ANN equalizer is then employed to compensate for the nonlinear electrical signal distortions. Finally, a decision circuit is placed at the end of the DSP unit to classify the equalized data ${\hat{X}}_{i}$ to its nearest symbol ${\hat{a}}_{i}=\left\{0,1\right\}$.

For all results presented in this section, the performance, efficiency, and effectiveness of the proposed ANN equalizer are investigated using three figures of merit, namely, the bit error rate (BER) performance of the overall communication system in the presence of noise, the computational cost per symbol required by the equalizer, and the memory usage requirements of the equalizer.

Firstly, the BER performance is calculated through Monte-Carlo simulations of the communication system described in Figure 4. Using a pseudo-random binary sequence (PRBS) $2^{18}-1$, a stream of $2^{17}$ unknown random data are generated and transmitted through the communication system. Then, to achieve better BER accuracy with a smaller number of required symbols, we estimate the BER from its corresponding quality factor Q. Under the Gaussian noise assumption, the BER is related to the quality factor by [1,2]:

(4) $$\;\mathrm{BER}=\frac{1}{2}erfc\left(\frac{Q}{\sqrt{2}}\right),$$

where $erfc\left(\cdot \right)$ denotes the complementary error function. The quality factor is obtained using the following formula [1,2]:

(5) $$Q=\frac{I_{1}-I_{0}}{\sigma_{1}+\sigma_{0}},$$

where $I_{r}$ and $\sigma_{r}$, $r\in \left\{0,1\right\},$ are the mean and the standard deviation of the received signal, respectively, and r stands for the type of the transmitted symbol (‘0′ or ‘1′). It is worth emphasizing that single-polarized signals are typically used in short-reach optical-fiber communication systems where polarization mode dispersion (PMD) effect is negligible. The metric of BER performance is therefore enough to analyze the overall system performance since the optical-fiber channel is deterministic. Note also that the impact of fiber dispersion can be characterized by the dimensionless parameter: $B^{2}\beta_{2}L$, where B  is the baud rate, L is the fiber transmission distance, and $\beta_{2}$ is the fiber dispersion coefficient. For the given baud rate, if the fiber dispersion $\beta_{2}$ and/or the distance L increases, the ISI effect increases, which degrades the transmission performance. On the other hand, if the dispersion is fixed, the feasible transmission distance for the given BER scales inversely with $B^{2}$. Throughout this section, we show the BER performance versus the received optical power. However, the received power and signal-to-noise ratio (SNR) can be related from the following considerations. The receiver adds shot noise and thermal noise whose variances are given by [2]:

(6) $$\;\sigma_{shot}^{2}=2qRP_{r}B_{e},$$

(7) $$\sigma_{thermal}^{2}=4K_{B}TB_{e}/R_{L},$$

where q is the electron charge, R is the responsivity, $P_{r}$ is the received power, $B_{e}$ is the 3 dB bandwidth of the receiver electrical LPF, $K_{B}$ is the Boltzmann constant, T is absolute temperature in Kelvin, and $R_{L}$ is the load resistance. The SNR (without including optical noise) is given then by

(8) $$SNR=\frac{R^{2}P_{r}^{2}}{\sigma_{shot}^{2}+\sigma_{thermal}^{2}}.$$

Thus, SNR is directly related to the received optical power.

Secondly, using the input–output relationship of the proposed ANN equalizer (i.e., Equation (1)), it can be shown that the computational complexity of the ANN equalizer scales as ~m×N per symbol, where m and N are the numbers of hidden layer’s nodes and equalizer’s taps, respectively. Thirdly, once the ANN equalizer is trained, its memory storage requirements are essentially limited to the number of its neural network weight parameters W. For an ANN equalizer comprising N input taps and a single hidden layer with m nodes, the total number of weights is $\left(mN+m\right)$, with $\left(mN\right)$ parameters corresponding to the weights connecting between the input and hidden layer, and m weight parameters assigned to the connection between the hidden and output layer, as illustrated in Figure 1. Note that each real number is stored in the memory as a floating-point number [35]. Since the ANN equalizer weights are generally real numbers, the memory storage requirement of the proposed ANN equalizer is $\sim m\left(N+1\right)$ floating-point numbers.

Unless otherwise specified, the proposed ANN equalizer is trained using the advanced training scheme, described in Section 2. The number of noisy dataset groups is n=10. In other words, the total number of training data used is $N_{tr}=10\times M^{N+2K}$ symbols, where M=2 for the OOK modulation format. A total of 87.5% of the training dataset is used for training, while 12.5% of the data is utilized as the validation set during each epoch. The maximum allowed number of training epochs is 1000, and the batch size is 256. The hidden and output activation functions of the ANN are rectified linear unit (ReLU) function and unity function, respectively. The training is performed at a received optical power of 0 dBm. The training process is set to terminate when the validation set accuracy stops improving for a certain number of epochs, or the maximum allowed training epochs is exceeded.

### 3.1. A 10-Gbaud Optical-Fiber Communication System

In this subsection, we study the performance of the proposed ANN equalizer to compensate for the distortions of an IMDD short-haul fiber communication system operating at baud rate of 10 GBaud with optical-fiber transmission distance L ranging from tens of kilometers up to a few hundred kilometers. Such a communication system emulates typical communication scenarios in the area of metro and media access networks.

Consider a communication scenario with optical-fiber transmission distance, i.e., optical-fiber length, L=140 km. Due to pulse broadening caused by the chromatic dispersion and the square-law effect of the DD, the BER at the receiver without equalization and in the absence of noise is obtained as $4\times 10^{-2}$. Using convergence analyses, the numbers of equalizer taps (i.e., the ISI span) and hidden-layer nodes required for this case are estimated as N=7 and m=6, respecitevly. For better readability, we refer to the artificial neural network equalizer (ANNE) consisting of N input nodes and m hidden layer’s nodes, and trained using the advanced training (AT) scheme as ANNE-AT{N,m}.

**Figure 5:**
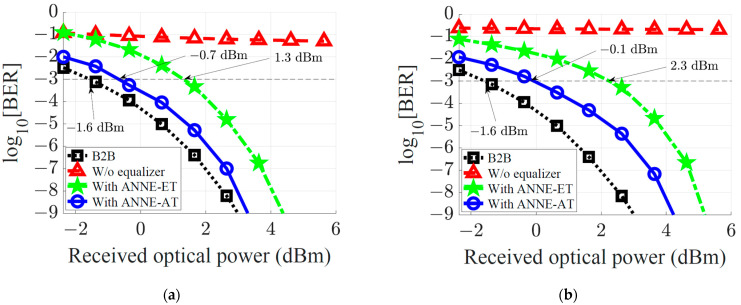
BER performance of the 10 Gbaud optical-fiber communication system versus received optical power with a noise-free EDFA. The optical-fiber transmission distance is (**a**) 140 km and (**b**) 200 km. The performance without equalizer, with ANNE-ET{7,6}, and with ANNE-AT{7,6} are compared to the B2B transmission case.

Figure 5a shows the BER performance obtained with the ANNE-AT{7,6} compared to the case with no equalization and the back-to-back (B2B) transmission case, versus a sweep of the received optical power at the transmission distance of 140 km. The ANNE-AT{7,6} effectively compensates for the nonlinear distortions of the system, extending transmission distance feasibility. It achieves the $\mathrm{BER}=10^{-3}$ at a received optical power of −0.7 dBm with ~0.9 dB power penalty relative to the B2B transmission case. To achieve the $\mathrm{BER}=10^{-9}$, the required received optical power is 3.3 dBm, with a minor power penalty of ~0.3 dB, compared to the B2B transmission case.

To evaluate the efficiency of the proposed advanced training scheme, we compare the performance of the ANNE-AT to another identical ANN-based equalizer but trained using the extensive training scheme introduced in [21]. For a fair comparison, all optimization parameters as well as the ANN configuration are kept the same during the training process. Such an ANN equalizer configuration that is trained using the extensive training (ET) scheme and comprises N input nodes and m hidden-layer nodes is denoted as ANNE-ET{N,m}. As can be seen from Figure 5a, using ANNE-ET{7,6}, the $\mathrm{BER}=10^{-3}$ is achieved at the received optical power of 1.3 dBm with a power penalty as high as ~2.9 dB relative to the B2B transmission case. At $\mathrm{BER}=10^{-9}$, the ANNE-ET{7,6} requires a received optical power of 4.4 dBm, with a ~1.4 dB power penalty compared to the B2B transmission case. Thanks to the proposed advanced training scheme, the ANNE-AT provides 2 dB and 1.1 dB power benefits over the ANNE-ET for achieving a BER of $10^{-3}$ and $10^{-9}$, respectively.

In Figure 5b, we explore the performance of the proposed ANNE at a longer fiber transmission distance of L=200 km. The same equalizer parameters N=7 and m=6 are utilized. The BER performance achieved using the ANNE-AT{7,6} versus received optical power are compared to that obtained with the no-equalization case, B2B transmission case, and the equalization using the ANNE-ET{7,6}. It is obvious that both ANNE-AT and ANNE-ET can extend the transmission distance up to 200 km. However, to obtain a $\mathrm{BER}=10^{-3}$, the ANNE-ET has a power penalty of 3.9 dB compared to the B2B case, while the ANNE-AT has only a 1.5 dB penalty. On the other hand, compared to the B2B case, to achieve a $\mathrm{BER}=10^{-9}$, power penalties using the ANNE-ET and ANNE-AT are 2.2 dB and 1.2 dB, respectively. In other words, a 2.4 dB and 1 dB power benefit is achieved by ANNE-AT, as opposed to ANNE-ET, to obtain a BER of $10^{-3}$ and $10^{-9}$, respectively. Comparing Figure 5a,b, we find that at a BER of $10^{-3},$ the performance benefit provided by the ANNE-AT over ANNE-ET increases at longer distances. That is because at a higher BER and/or longer distances, the impact of noise is more severe, and the advanced training of ANNE-AT under the noisy environment helps to achieve the better performance. Note that for a specific transmission distance, the BER is determined from the following factors: (i) ISI effect due to dispersion, which can be mitigated using the ANNE-AT equalizer provided that the number of taps is larger than the ISI span of the channel, (ii) channel noise that includes shot noise and thermal noise, and (iii) received optical power which depends on the transmitted power and fiber loss. The main purpose of the ANNE-AT equalizer is to mitigate the nonlinear distortions caused by fiber dispersion. The B2B results shown in Figure 5a,b are the minimum BER obtainable for the given received signal power and noise power levels. From these figures, it can be seen that ANNE-AT performance is quite close to the B2B case.

**Figure 6:**
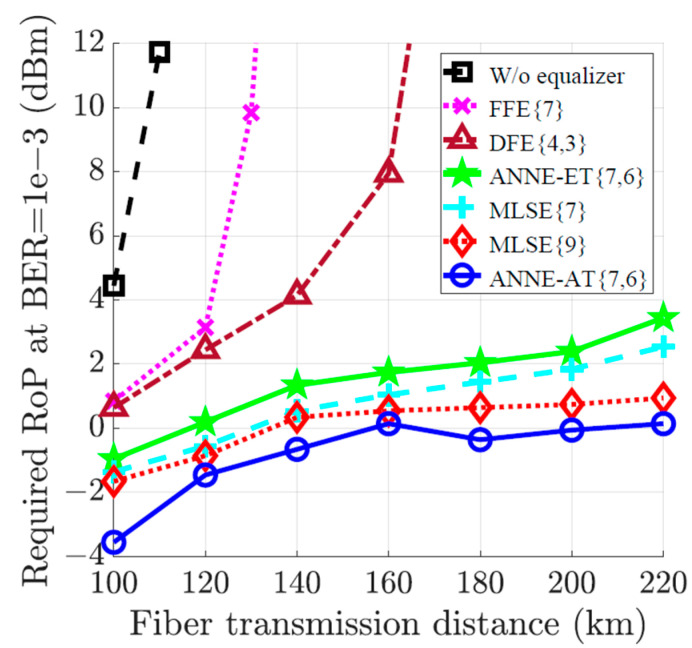
Received optical power (RoP) required to achieve $\mathrm{BER}=10^{-3}$ versus fiber transmission distance for the 10 Gbaud optical-fiber communication system. The equalization at the receiver is performed using the FFE{7}, DFE{4,3}, ANNE-ET{7,6}, MLSE{7}, MLSE{9}, and ANNE-AT{7,6}.

To further demonstrate the efficiency of the proposed ANNE-AT, three types of common equalization schemes including feed-forward equalizer (FFE), decision feedback equalizer (DFE), and maximum likelihood sequence estimator (MLSE) are considered for comparative analysis. The received optical power required to achieve $\mathrm{BER}=10^{-3}$ versus a sweep of the fiber transmission distance is shown in Figure 6. Particularly, we compare the BER performance of the ANNE-AT to FFE$\left\{7\right\}$, DFE$\left\{4,3\right\}$, ANNE-ET, MLSE$\left\{7\right\}$, and MLSE$\left\{9\right\}$, where $\left\{\cdot \right\}$ refers to the number of FFE taps or the memory size of the MLSE. In the case of DFE, $\left\{\cdot ,\cdot \right\}$ denotes the numbers of its feed-forward and feed-backward taps, respectively. Note that the parameters of the ANNE-AT and ANNE-ET are fixed to N=7 and m=6 for all distance cases shown in Figure 6. For a fair comparison, we use the advanced training scheme to train both the FFE and DFE equalizers. However, the MLSE is trained using the extensive training scheme. Note that the MLSE training process aims to construct a look-up table that considers all the distorted noise-free sequences that may be received after the channel transmission. Once trained, the MLSE estimates the transmitted sequence by comparing the received distorted sequence with all look-up table entries and selects the sequence with highest probability. Therefore, it is impractical to train the MLSE using the advanced training scheme as this will significantly enlarge the size of its look-up table, leading to an infeasible equalization time.

As can be seen from Figure 6, the feasible transmission distance is limited to less than 100 km if no equalization is used. The linear equalizers including the FFE and DFE can barely extend the feasible transmission distance to around 100 km. They fail to compensate for the nonlinear distortions at longer transmission distances. However, nonlinear equalizers including the proposed ANNE-AT, the ANNE-ET, and the MLSE can provide a BER performance benefit, significantly extending the transmission distance feasibility. Clearly, the ANNE-AT shows superior BER performance over all other nonlinear equalizers. The power benefit of the ANNE-AT over ANNE-ET ranges from 1.6 dB to 3.3 dB with an average power benefit of 2.3 dB. Owing to the strength of the advanced training scheme, the ANNE-AT also provides a 1.6 dB power benefit, on average, as opposed to the MLSE{7}, and a slight power benefit over the MLSE{9}.

In terms of the overhead cost and memory usage required for equalization, Table 1 tabulates the computational complexity per symbol and the memory storage requirements of the FFE, DFE, ANNE-ET, and MLSE compared to the proposed ANNE-AT. The minimum overhead equalization cost and memory requirements are achieved by the FFE and DFE. However, as can be seen from Figure 6, both the FFE and DFE provide poor compensation performance benefit because of their limited ability against nonlinear system distortions. Particularly, the computation cost per symbol of the FFE{N} and DFE{$N_{1}$,$N_{2}$} scales linearly as ~N and $\sim \left(N_{1}+N_{2}\right)$, where N is the number of FFE taps, and $N_{1}$ and $N_{2}$ are the numbers of feed-forward and feed-backward taps of the DFE, respectively [36]. During equalization, both the FFE and DFE also need to store the values of their tap weights, where the number of FFE and DFE weight parameters is equal to their total number of taps [36]. The memory requirements of the FFE{N} and DFE{$N_{1}$,$N_{2}$} therefore scale as ~N and $\sim \left(N_{1}+N_{2}\right)$ floating-point numbers, respectively. The computational complexity per symbol and memory storage requirements of the ANNE-AT (as well as the ANNE-ET) scale as ~mN and $\sim m\left(N+1\right)$, respectively. However, the computational cost of the MLSE scales exponentially as ∼$2^{N}$ per symbol, where N  is the memory size of the MLSE [36]. In addition, the MLSE requires huge memory storage during the equalization process, because it needs to store all entries of its look-up table to compare them with the received distorted sequence and determine the most probable transmitted sequence [36]. It thus follows that the memory usage requirements of the MLSE scale as high as the size of its look-up table, i.e., $\sim 2^{N}$ floating-point numbers.

From Table 1, to obtain the results provided in Figure 6, the FFE{7}, DFE{3,4}, MLSE{7}, and MLSE{9} particularly require 7, 7, 128, and 512 multiplication operations per bit, respectively, whereas the ANNE-AT{7,6} and ANNE-ET{7,6} need only 42 multiplication operations per bit. During the equalization process, the ANNE-AT{7,6} and ANNE-ET{7,6} also require a memory storage of 48 floating-point numbers, as opposed to 7, 7, 128, and 512 floating-point numbers needed to be stored by the FFE{7}, DFE{3,4}, MLSE{7}, and MLSE{9}, respectively. Hence, among all tested nonlinear equalizers that provide the required performance benefit, the ANNE-AT is the most efficient equalizer from the performance, computation complexity, and memory requirement points of view.

**Figure 7:**
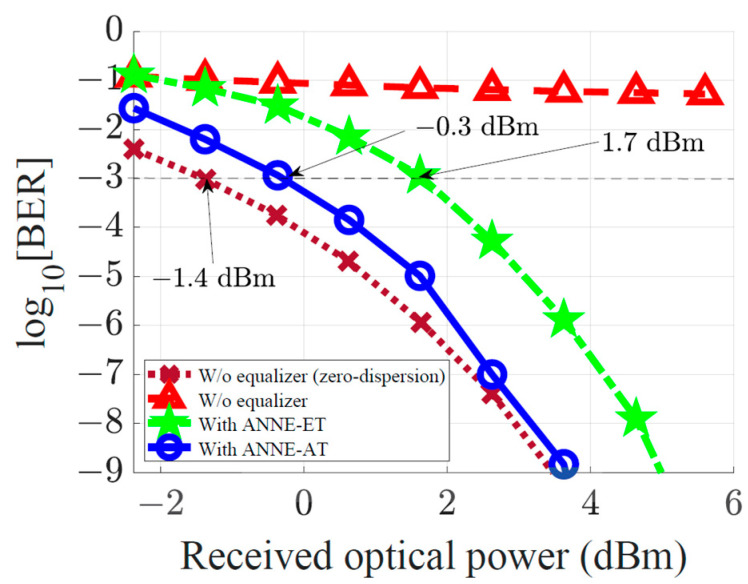
BER performance of the 10 Gbaud optical-fiber communication system versus received optical power with a noisy EDFA whose noise figure is 4.77 dB. The optical-fiber transmission distance is 140 km. The performance without equalizer, with ANNE-ET{7,6}, and with ANNE-AT{7,6} are compared to the zero-dispersion transmission case.

So far, we have assumed a noise-free EDFA. However, in addition to amplifying the optical signal, actual EDFAs add optical noise to the signal due to spontaneous emission effect [1,2]. The interaction between this added optical noise with the fiber dispersion and square-law detection of the DD results in further BER performance degradation. To investigate this degradation, we consider the 140 km communication scenario with a noisy EDFA having a noise figure $N_{f}=4.77\;\mathrm{dB}$. The ANNE-AT model used to obtain the results of Figure 5a is re-trained to include the effect of the EDFA optical noise. Figure 7 plots the BER performance of the overall system including the EDFA noise effect versus the received optical power. We compare the BER performance achieved using the ANNE-AT{7,6} with that obtained in the no-equalization case, equalization using the ANNE-ET{7,6}, and the zero-dispersion transmission case. Notice that the minimum BER that can be achieved in this case is not the BER performance of the B2B transmission case, which does not include the effect of the EDFA optical noise. Rather, the reference BER that we seek to converge to, in this case, is the BER performance of the 140 km transmission over a non-dispersive (i.e., $\beta_{2}=0\;{\mathrm{ps}}^{2}/\mathrm{km}$) optical-fiber link, i.e., the zero-dispersion transmission case (‘crosses’ in Figure 7).

As can be seen from Figure 7, the overall system performance slightly deteriorates compared to the case of noise-free EDFA (see Figure 5a). The ANNE-ET obtains $\mathrm{BER}=10^{-3}$ and $10^{-9}$ at received optical powers of 1.7 dBm and 5.0 dBm, respectively, as opposed to the 1.3 dBm and 4.4 dBm needed in the case of noise-free EDFA. In other words, the EDFA noise affects the performance obtained using the ANNE-ET by a 0.4 dB and 0.6 dB at BER of $10^{-3}$ and $10^{-9}$, respectively. As compared to the zero-dispersion performance, the ANNE-ET requires ~3.1 dB and ~1.5 dB power penalties to obtain the $\mathrm{BER}=10^{-3}$ and $10^{-9}$, respectively. On the other hand, the ANNE-AT shows superior performance that is quite close to the zero-dispersion transmission case, owing to the inclusion of the system noise effect during its training process. Particularly, the presence of the EDFA optical noise reduces the BER performance achieved utilizing the ANNE-AT by 0.4 dB at both the $\mathrm{BER}=10^{-3}$ and $10^{-9}$ levels (see Figure 5a and Figure 7). Compared to the zero-dispersion transmission case, the ANNE-AT requires power penalties of as low as ~1.1 dB and ~0.2 dB to achieve $\mathrm{BER}=10^{-3}$ and $10^{-9}$, respectively. Moreover, the proposed ANNE-AT outperforms the ANNE-ET by power benefits of ~2 dB and ~1.3 dB for achieving a BER of $10^{-3}$ and $10^{-9}$, respectively. Comparing this with the case of no optical noise (Figure 5a), we find that the inclusion of the EDFA noise effect does not change the average power benefit of the proposed ANNE-AT over the ANNE-ET.

### 3.2. A 28-Gbaud Optical-Fiber Communication System

This subsection investigates the performance of the proposed equalizer to mitigate the impairments induced at a higher transmission data rate. Particularly, we discuss its performance to mitigate the distortions of an IMDD short-reach fiber communication system operating at a baud rate of 28 Gbaud with optical-fiber length L ranging from a few meters up to a few tens of kilometers. Such communication systems are widely deployed in typical communication scenarios of varied applications, e.g., data center networks (DCNs) and cloud radio access networks (C-RANs). They can exist as 28 Gbaud serial links or as 4×28 Gbaud parallel links, implementing a data rate of 100 Gbaud. Note that optical amplifiers are not typically used in these systems due to short transmission distances. Therefore, for all results shown in this subsection, we consider the exact system setup given in Figure 4 excluding the EDFA block, which is not required here.

**Figure 8:**
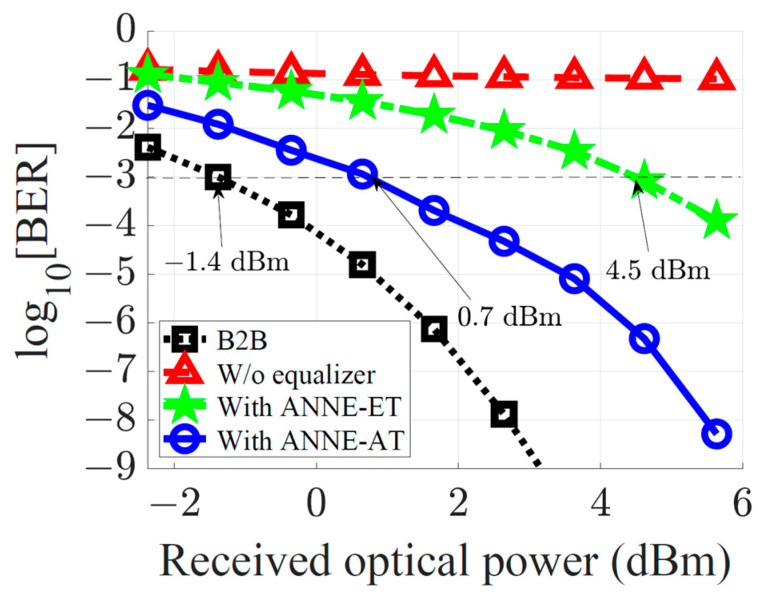
BER performance of the 28 Gbaud optical-fiber communication system versus received optical power. The optical-fiber transmission distance is 20 km. The performance without equalizer, with ANNE-ET{7,6} and with ANNE-AT{7,6} are compared to the B2B transmission case.

In Figure 8, we show the BER performance obtained at a fiber transmission distance L=20 km versus received optical power. The BER achieved using the ANNE-AT{7,6} is compared to the system performance at no equalization, the B2B transmission case, and equalization utilizing the ANNE-ET{7,6}. It is clear that increasing the data rate significantly deteriorates the system performance, resulting in an infeasible transmission in the case of no equalization. However, the ANNE-AT can be utilized to improve dispersion tolerance, extending the feasible transmission distance. Compared to the B2B transmission case, the ANNE-AT is shown to achieve the $\mathrm{BER}=10^{-3}$ with a 2.1 dB power penalty, whereas the ANNE-ET has a power penalty of 5.9 dB. In other words, the ANNE-AT outperforms the ANNE-ET with a ~3.8 dB power benefit to achieve $\mathrm{BER}=10^{-3}$ in this case.

**Figure 9:**
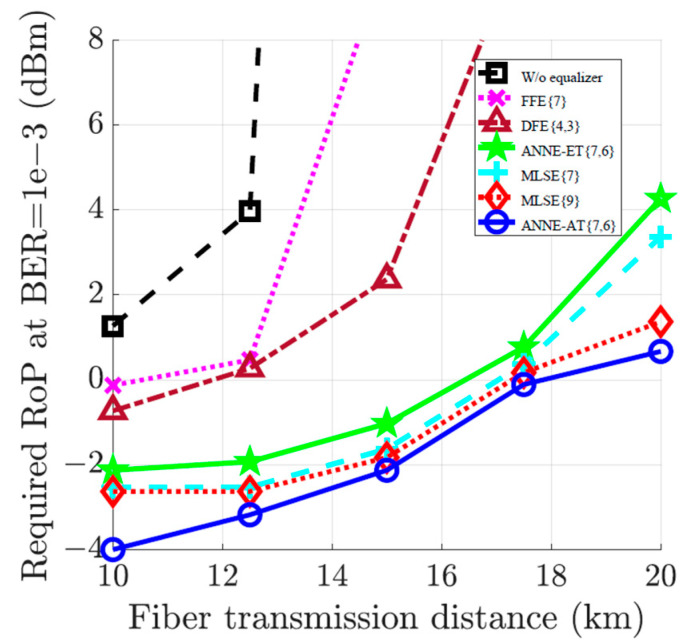
Received optical power (RoP) required to achieve $\mathrm{BER}=10^{-3}$ versus fiber transmission distance for the 28 Gbaud optical-fiber communication system. The equalization at the receiver is performed using the FFE{7}, DFE{4,3}, ANNE-ET{7,6}, MLSE{7}, MLSE{9}, and ANNE-AT{7,6}.

To demonstrate efficiency of the ANNE-AT compared to other equalizers, Figure 9 plots the received optical power required to achieve $\mathrm{BER}=10^{-3}$ versus the fiber transmission distance. The compensation performance of the ANNE-AT{7,6} is compared to the performance of the FFE{7}, DFE{4,3}, MLSE{7}, and MLSE{9}, as well as the ANNE-ET{7,6}. As can be seen, the proposed ANNE-AT provides an average power benefit of 1.7 dB and 1.2 dB compared to the ANNE-ET and MLSE{7}, respectively, while slightly outperforming the MLSE{9}. Moreover, the ANNE-AT significantly reduces the overhead cost and memory storage required by the MLSE during equalization (see Table 1). A total of 42 multiplication operations per bit are required by the ANNE-AT as opposed to 128 and 512 in cases of the MLSE{7} and MLSE{9}, respectively. In addition, the MLSE{7} and MLSE{9} require a memory storage of as high as 128 and 512 floating-point numbers as opposed to only 48 floating-point numbers needed to be stored by the ANNE-AT.

Although we considered only the case of on–off keying (OOK) modulation format throughout this paper, the proposed ANN equalizer can be easily applied to higher modulation formats, e.g., pulse amplitude modulation (PAM) 4 or PAM 8. For instance, if PAM 4 were considered, the output of the ANN equalizer would apply on symbols, rather than bits, and vary between four states instead of the current two states of the OOK. However, we defer this study to future work.

## 4. Conclusions

We propose an improved artificial neural network (ANN)-based equalization method. It effectively compensates for the nonlinear impairments associated to the received electrical signal in the short-haul fiber-optic communication systems based on intensity modulation and direct photo-detection. The proposed equalizer consists of a single hidden neural network layer which significantly reduces the overhead computational cost and memory storage requirements of the equalization step. The performance and efficiency of the proposed equalizer are demonstrated through 10- and 28-Gbaud short-haul fiber communication systems. Although the minimum overhead equalization cost and memory usage are achieved by the linear feed-forward equalizer (FFE) and decision feedback equalizer (DFE), they both provide poor compensation performance benefit due to their inability to mitigate nonlinear system distortions. The proposed ANN equalizer, on the other hand, provides the required nonlinear compensation performance benefit with slightly higher computational cost and memory usage. The proposed ANN equalizer with the integrated advanced training scheme also outperforms the maximum likelihood sequence estimator (MLSE), achieving an average power benefit of more than 1 dB, when the same number of taps and memory size is considered. Furthermore, the proposed ANN equalizer requires much less computational cost and memory storage. Its cost and memory usage scale linearly with the number of input nodes N and with the number of hidden-layer nodes m, whereas the computational cost and memory usage of the MLSE scale exponentially with the channel memory size N. Note that the power consumption of the proposed equalizer is not discussed in this paper. The power consumption scales directly with the computational cost and memory usage of the equalizer. In other words, the ANN equalizer should significantly reduce the high power consumption required by the MLSE as well. It is also worth emphasizing that the proposed ANN equalizer can easily be implemented in a field-programmable gate array (FPGA) integrated circuit/kit and attached to any available/commercial digital signal processing (DSP) unit without any hardware constraints or limitations.

## Figures and Tables

**Table 1:** A comparison of the computational complexity and memory storage requirements during equalization.

Equalizer	Per Symbol Computational Cost	Memory Storage ^1^
FFE{N}	N	N
DFE{$N_{1}$,$N_{2}$}	$N_{1}+N_{2}$	$N_{1}+N_{2}$
ANNE-AT/ET{N,m}	mN	$m\left(N+1\right)$
MLSE{N}	$2^{N}$	$2^{N}$

^1^ Memory storage is given in terms of the required floating-point numbers to be stored.

## Data Availability

Data underlying the results presented in this paper are not publicly available at this time but may be obtained from the authors upon reasonable request.
